# The Polysaccharidic Nature of the Skeleton of Marennine as Determined by NMR Spectroscopy

**DOI:** 10.3390/md21010042

**Published:** 2023-01-04

**Authors:** Ilhem Zebiri, Boris Jacquette, Nellie Francezon, Mickaël Herbaut, Amina Latigui, Sullivan Bricaud, Réjean Tremblay, Pamela Pasetto, Jean-Luc Mouget, Jens Dittmer

**Affiliations:** 1 Laboratoire Biologie des Organismes, Stress, Santé, Environnement (BiOSSE), Le Mans Université, Av. Olivier Messiaen, 72085 Le Mans, France; 2 Institut des Molécules et Matériaux du Mans (IMMM), UMR CNRS 6283, Le Mans Université, Av. Olivier Messiaen, 72085 Le Mans, France; 3 Institut des Sciences de la Mer, Université du Québec à Rimouski, 310 Allée des Ursulines, Rimouski, QC G5L 3A1, Canada

**Keywords:** marennine, *Haslea ostrearia*, microalgae, diatoms, polysaccharides, blue pigment, biomolecular NMR

## Abstract

The water-soluble blue–green pigment marennine, produced and partly excreted by the diatom *Haslea ostrearia*, and known for a long time for its role in the greening of oysters, was isolated from the culture medium, purified, and analyzed by Nuclear Magnetic Resonance (NMR) in order to gain insight into its chemical structure. The spectra show mainly carbohydrates of a complex composition, apparently highly branched, and with a mass in the order of 10 kDa. There are, in addition, some signals of aliphatic and, much weaker, aromatic groups that present aglycons. The latter might be responsible for the color. These carbohydrates are always associated with the blue–green color and cannot be separated from it by most treatments; they are interpreted as constituting the frame of the pigment. NMR after hydrolysis identifies the most abundant monosaccharides in marennine as galactose, xylose, mannose, rhamnose, and fucose.

## 1. Introduction

**Figure 1:**
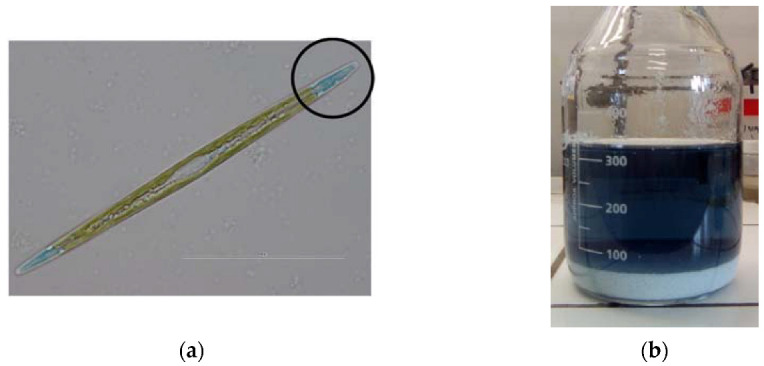
(**a**) Diatom *Haslea ostrearia* with a concentration of marennine in the apices. (**b**) Solution of extracellular marennine (EMn) after initial purification by precipitation and re-solubilization in formic acid.

The greening of oysters and other marine invertebrates in ponds has been observed for a long time, and it was described first by Sprat in 1669 [1]. Sprat’s description was based on observations close to Colchester at the British coast of the North Sea, but the phenomenon occurs in fact at the coasts of seas almost all over the world. A particular place is the Bay of Marennes-Oléron at the French Atlantic Coast, where these blue–green oysters have always been considered as a culinary particularity (“Verte de Claire” or “Fine de Claire Verte”), presenting a certain economical added value. As the ponds are also colored, it was concluded that the colorant is not produced by the oysters themselves. It turned out that a microalga from the class of *Bacillariophyceae*, *Haslea ostrearia*, produces and transiently stores in its apices a blue–green pigment (Figure 1a) [2,3,4], then called marennine after its most prominent occurrence [5], which is excreted into the seawater. Marennine is usually denoted as pigment in most biological studies, although it is strictly speaking a colorant, being highly water soluble and occurring in solution in marine environments. Pouvreau et al. [6] distinguish two forms, the extracellular (in the following denoted EMn) and the intracellular form (IMn), that differ in their molecular mass and UV–Visible spectra. The oysters filter the seawater using their gills, select particles and thus feed on microalgae, including *Haslea* cells [7]. It has, however, been demonstrated that the greening of oysters is mainly the consequence of water filtration, which results in the fixation of marennine on gills [8].

Apart from the greening of oysters, marennine has some interesting potential applications, the most obvious ones related to being a colorant. All the more, as the color shifts with lower pH to blue [9], a color that is rare in the spectrum of natural food colorants [10]. In a very different direction, studies have shown that marennine has also antibiotic effect against *Bacillus cereus*, *Staphylococcus epidermidis*, *S. aureus*, *Vibrio anguillarum*, *V. aestuarianus*, and *V. splendidus* [11,12,13,14], some of the most frequent pathogens in aquaculture. A straightforward application would thus be the protection of aquacultures against these bacteria and possibly other pathogens.

In the regard of its properties and the long history of research on marennine, it is surprising that there is relatively little known about its chemical structure. In fact, during the more than 100 years of study of its structure, nearly any class of molecules that can be found in organisms has been suggested. The earliest hypothesis (1845) attributed the color to metallic ions—in particular, copper [15,16]. Ryder (1884) [17], Bocat (1907) [9] and later Robert and Hallet (1981) [18] suggested that the pigment or part of it was a protein, like in cyanobacteria. A relation to chlorophyll or its degradation products—suggesting itself by the color—was proposed by Bachrach in 1935 [19] and has been resumed several times (for example, by Genvès et al. [20]). Ranson, however, found indications that marennine was a carotenoide [21]. Interesting with respect to the present work is the hypothesis of Neuville and Daste [22], who concluded from the analogy in the pigment production as a response to a limitation in nitrate with the anthocyanin production of some higher plants that marennine might be an anthocyanidin, thus a flavonoid pigment usually occurring together with a saccharide rest as anthocyanin. Additionally, Pouvreau et al. concluded after a series of tests against various classes of molecules that marennine could be a polyphenol [6]. There has been less discussion about the size of the molecule, although also this question is not answered definitely. It is considered to be clear that marennine is a macromolecule. While Vandanjon et al. conclude from ultrafiltration a polydispersity with a maximum of the distribution in the order of 3–7 kDa [23] in the extracellular form, Pouvreau et al. find 10–12 kDa by SDS-PAGE, 7 (EMn) and 8 (IMn) kDa by gel-filtration chromatography and a monodisperse mass of 9894 Da (EMn) and 10,751 Da (IMn) by mass spectrometry [6]. Many of the hypotheses about the structure cited above are based on biochemical tests, which probe only one aspect of the sample and which are therefore prone to false results in the case of an imperfect purification or if the molecule is heterogeneous and very complex. Nuclear Magnetic Resonance (NMR) of solutions, in contrast, provides signals of all hydrogens or carbons in the sample that are part of molecules smaller than the order of 100 kDa in—with some limitations—proportion to their abundance. It has been very successful in the determination of the spatial structure of proteins and is in general considered as one of the most important tools in the analysis of organic molecules. We have previously presented indications by NMR that marennine could be, in part, a polysaccharide [11], based on data acquired on an NMR spectrometer with a relatively low field and corresponding sensitivity and resolution limits.

Polysaccharides are the major component of extracellular polymeric substance (EPS), excreted by many prokaryote and eukaryote microorganisms (e.g., [24,25]). So do microalgae in general and diatoms in particular, for which EPS may facilitate cell adhesion to a surface or migration in a biofilm or a sediment, ensure cell protection from chemical, osmotic or desiccation stress or constitute a reserve of nutrients [26,27]. Exopolysaccharides typically exhibit a complex chemical structure. Their properties—in particular, their biological activities—make them interesting for industrial and agricultural applications, and marennine is a good example of this [11]. For a review of this emerging field, see [28]. Recently, Bélanger et al. [29] developed a method to separate other polysaccharide fractions from that of marennine and found evidence by FTIR spectroscopy that these polysaccharides are sulfated and discussed their relation to fucoidan.

The present study characterizes marennine in more detail by NMR, determines the monosaccharide constituents after hydrolysis and discusses the results with respect to previous findings and the nature of the chromophore.

## 2. Results

### 2.1. NMR of the Extracellular Form of Marennine (EMn)

**Figure 2:**
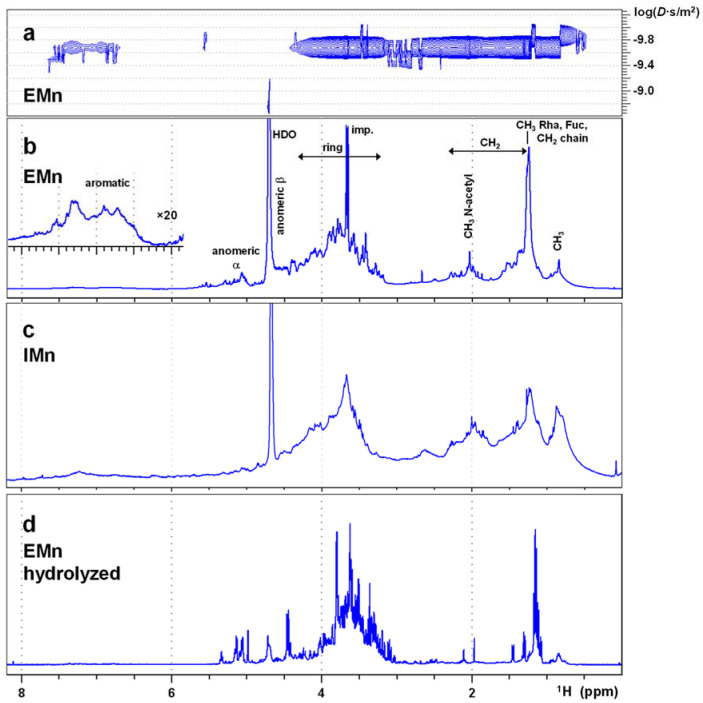
(**a**) ^1^H DOSY and (**b**) single pulse ^1^H spectrum of extracellular marennine (EMn) in D_2_O. The aromatic part is scaled up by a factor of 20 in (**b**). (**c**) ^1^H spectrum of intracellular marennine (IMn) and (**d**) hydrolyzed EMn.

**Figure 3:**
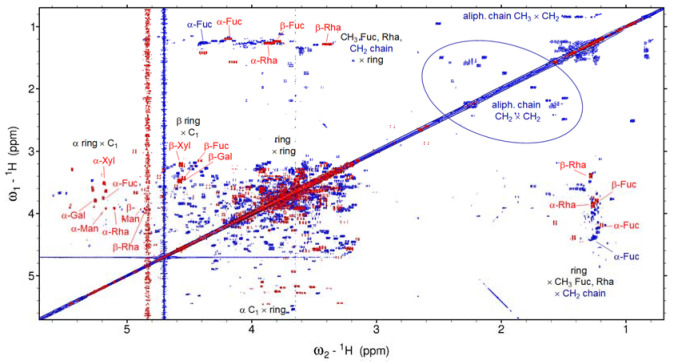
COSY spectra of native (dark blue/blue) and hydrolyzed (dark red/red) external marennine EMn. The spectrum of hydrolyzed EMn is shifted by +0.12 ppm in both dimensions. Some assignments are indicated: blue: native, red: hydrolyzed and black: general regions.

A ^1^H single pulse experiment of extracellular marennine (EMn) yields a complex spectrum without resolved lines, covering mainly the region between 3 and 4 ppm, extending up to 5.5 ppm (Figure 2b). This is the typical region of the ring protons of carbohydrates. Furthermore, there are signals in the aliphatic range and a weak band in the aromatic range. The blue contour lines of Figure 3a represent the COSY spectrum of EMn. The signals of the 3–4 ppm region have cross peaks within this region to aliphatic signals and, most important, to signals ranging beyond the water line up to 5.7 ppm. Signals in this area are characteristic for the rings of carbohydrates, including the anomeric protons at 4.3–4.9 (β-conformation) and 4.9–5.6 ppm (α). The rings, including anomerics, are confirmed by HSQC (Figure S1a,c) and, furthermore, CH_2_OH groups at (59–66) ppm for ^13^C (Figure S1a). The COSY shows cross-peaks of ring hydrogens to methyl groups (1.1 ppm for ^1^H, HSQC correlation in Figure S1b). Marennine thus contains methyl monosaccharides.

The DOSY spectrum of marennine shows, apart from some sharp lined impurities, a relatively homogeneous diffusion coefficient of 10^−9.69^ m^2^/s = 2.0 × 10^−10^ m^2^/s, corresponding to a molecular size of the order of 10 kDa (Figure 2a). Also the aromatic signals have the same diffusion coefficient, indicating that they belong to the same molecule. The mass is in agreement with the results of Pouvreau et al. [6] obtained by mass spectrometry (9893 Da) and gel-filtration chromatography (about 7 kDa), while it is somewhat above the 3–7 kDa found by Vandanjon et al. by ultrafiltration [23]. Note that the DOSY experiment is not very precise, and the interpretation of the diffusion coefficient in terms of molecular mass adds further uncertainty. Moreover, the experiment has only low resolution, so that the measured diffusion coefficient can be the average of a distribution. In fact, with different preparations, we observed a certain variation beyond the experimental error, rather indicating a polydispersity, which is moreover common in polysaccharides. Mass spectrometry of our preparations, although tried with different techniques by different laboratories, did not give any results, the reasons for which might also be polydispersity and a negative charge of the macromolecule (polyanion).

Compared to many typical polysaccharides, the spectra of marennine show a high complexity with, e.g., some hundreds of resolvable COSY signals. Even when taking into account that some of them might be impurities due to imperfect purification, marennine must thus consist of several types of monosaccharides with different glycosidic linkages. The spectra have therefore (and for reasons of sensitivity) been acquired on a high field (950 MHz) spectrometer equipped with a cryogenic probe. Despite the use of this instrument, even many anomeric signals are not very well defined (Figure S1c), with heterogeneity in the HSQC signal intensities. Overall, it is possible to recognize about 30 different HSQC signals in the anomeric region, about half of them in the chemical shift regions of α- and β-conformation, respectively. Thirty typical monosaccharides already correspond to a mass of more than 5 kDa, compared to an overall molecular mass of the order of 10 kDa. This dispersion of signals of HSQC and the high number of COSY signals indicate a high heterogeneity of monosaccharides and glycosidic bonds and probably a molecular polydispersity with some characteristic motifs and only a few repetitions. Glycosidic bonds, but also steric interactions, change the chemical shifts compared to monosaccharides [30], which makes the analysis of the overlapping signals peak by peak in this complex spectrum virtually impossible. Only few signals could be assigned—in particular, the intense signals of a spin system that is attributed to β-xylose at the nonreducing end (Figures S1 and S2). This, if not being an experimental artifact due to slower relaxation, indicates a certain degree of branching with β-xylose at the termini. Furthermore, these β-xylose signals show extensive dispersion in both dimensions, well visible, for example, in the HSQC-TOCSY cross-peaks to C5 and H2/H5′’ (Figure S2), another sign of the heterogeneity of the molecule.

The signal group at 2 ppm in the proton spectrum (Figure 2b) is characteristic for the methyls of acetyl groups. It indeed shows the corresponding correlation signals (HSQC: x 22.1–22.7 ppm, Figure S1b; HMBC: x 173.2–174.4 ppm, carbonyl). Marennine in nondeuterated water shows in addition a group of small (water-exchangeable) proton signals at 7.8–8.6 ppm, which correlate with the proton signals of the ring region and with ^15^N around 120 ppm in a SOFAST-HSQC. They are thus most likely stemming from the amide H-N of N-acetylated amino sugars of unknown types in several conformations—however, in lower abundance.

There are additional signals in the aliphatic region of spectra of marennine (0.8–2.7 ppm ^1^H) that belong to the same diffusion coefficient. Some of them stem from the methyl group of fucose (15.0–16.0 ppm ^13^C, 1.18–1.29 ppm ^1^H) and rhamnose (16.3–17.0 ppm ^13^C, 1.18–1.37 ppm ^1^H, Figure S1), vide infra. The majority of the other aliphatic signals can be attributed to two different saturated fatty chains, bound by esters or perhaps linked to the N-acetyl groups discussed above. In particular, the intense HSQC signal at 28.8 ppm × 1.24 ppm indicates a longer CH_2_ chain. The presence of such a hydrophobic element will likely have an influence on the spatial structure of marennine in water.

### 2.2. NMR of the Intracellular Form of Marennine (IMn)

The proton spectrum of IMn resembles that of EMn but is not identical (Figure 2c). The ^1^H signal at 1.24 ppm is significantly smaller. It consists of the methyl groups of rhamnose and fucose, and the part with longer CH_2_ chains is much smaller than for EMn (HSQC, not shown). Instead, there are many more (different) methyl groups (0.8–0.9 ppm). Perhaps the longer chain in EMn is related to the excretion of marennine, possibly via vesicles [31]. Furthermore, there seem to be less monosaccharides in the α-conformation. Apart from these aspects, the differences between IMn and EMn are within the variations of different preparations of EMn or IMn.

### 2.3. NMR of EMn after Acidic Hydrolysis

Due to the complexity of the spectrum of the entire molecule, marennine was hydrolyzed by incubating in 0.5 M HCl at 95 °C for 2 h. These harsh conditions are necessary to successfully hydrolyze marennine [32], as controlled by the ^1^H spectra of the fraction that remained in the aqueous phase after the reaction (Figure 2d); all signals become much sharper due to the smaller size of the molecules. The diffusion coefficient increases to about 5 × 10^−10^ m^2^/s according to the DOSY experiment (Figure S3), corresponding to the order of the typical molecular mass of monosaccharides, about 200 Da. The increase in transverse relaxation times not only leads to a better resolution but also to higher signal intensities of experiments with longer pulse sequences.

The spectra now show virtually exclusively signals that are typical for carbohydrates. The residuals of the other groups thus have remained in the precipitate. The anomeric signals have shifted in the carbon dimension by, on average, −7 ppm, due to the cleavage of the glycosidic bonds (Figure S1) [33]. Only three small residual signals are left beyond 100 ppm (not shown). The aromatic signals in the ^1^H 1D spectrum and the aliphatic–aliphatic cross-peaks in the COSY spectrum (red contour lines in Figure 3) have virtually vanished, probably due to a drop of these groups into the precipitate and a faster relaxation of the residual bigger molecule fragments. Dispersion of chemical shifts due to different glycosidic bonds and secondary structure is expected to disappear, which simplifies the spectrum. On the other hand, isolate monosaccharides are always present in both α- and β-conformations and sometimes also as both pyranose and furanose forms, which increases the number of signals. The spectra are still quite complex but overall significantly simplified after hydrolysis, and the majority of the resonances can be assigned.

The spin systems are identified on the basis of all stronger HSQC signals and the COSY signals, the patterns of which allow for a rough estimation of the *J*-couplings. These reflect the dihedral angle between neighboring hydrogens and facilitate the distinction between axial and equatorial conformations.

**Figure 4:**
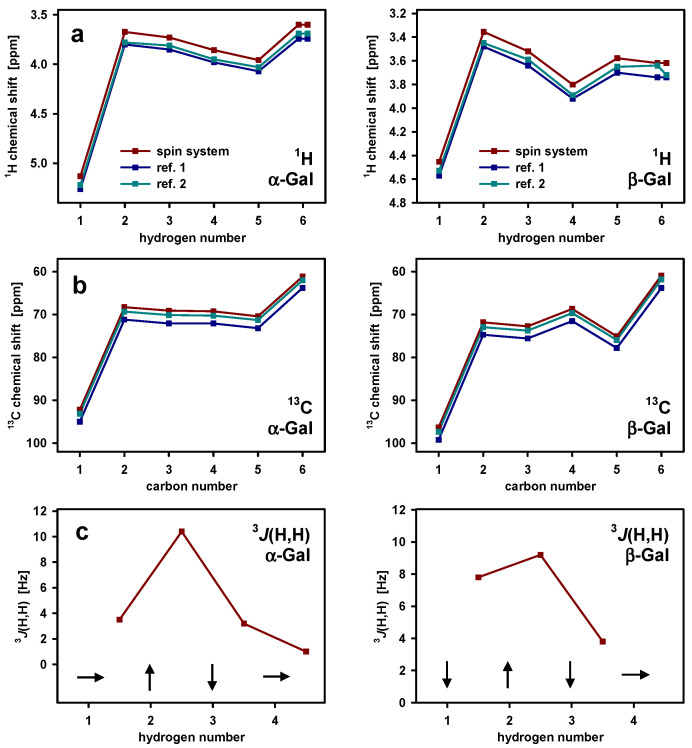
(**a**) ^1^H and (**b**) ^13^C chemical shifts of two spin systems of hydrolyzed extracellular marennine EMn (red) compared with the literature values of α- and β-galactose taken from [34] (ref. 1, blue) and [30] (ref. 2, green). (**c**) *J*-couplings between neighboring hydrogens as estimated from the COSY peak patterns and hydrogen orientation (axial up, down, or equatorial) derived from them.

Figure 4a,b show ^1^H and ^13^C chemical shifts and Figure 4c approximate values of the *J*-couplings for two of the identified spin systems. The sequence of the *J*-couplings corresponds to β-galactose. The chemical shifts show the same pattern as the literature values of β-galactose—however, with a constant deviation of about −0.12 [34] and −0.07 [30] ppm, respectively, probably due to the different pH and temperatures. These differences are, within some variations, the same for all found monosaccharides, including the methyl hydrogens (Figure S4). The HSQC spectrum allows for the identification of the corresponding carbon signals in conjunction with an HMBC, which has the further advantage of narrower lines due to the absence of decoupling limitations. Figure 4b shows the corresponding carbon chemical shift pattern; the deviation to the references are −2.8 [34] and −1.0 [30] ppm, respectively. In this way, almost all hydrogen and carbon signals of the α- and β-conformations of galactose, xylose, rhamnose, and fucose, as well as of the α-conformation of mannose, can be identified. For β-mannose, having a low concentration, only some signals are found. See Figure S4 for the corresponding *J*- and chemical shift patterns. Note that the presence of both conformers in the hydrolyzed marennine does not mean that both are also present in native marennine. We observe just the natural equilibrium of the two conformations of the monosaccharides in solution.

**Figure 5:**
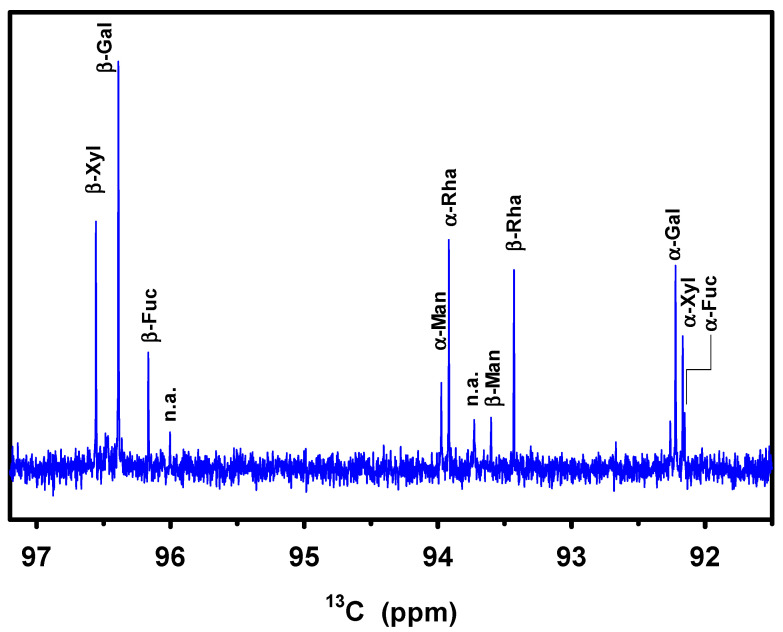
Anomeric region of a ^13^C DEPT spectrum of hydrolyzed extracellular marennine EMn. (n.a.: not assigned).

The anomeric region of a 1D DEPT ^13^C spectrum of the hydrolyzed sample that has optimal spectral resolution is used for quantification (Figure 5). The difference due to different J-couplings (160 Hz for α- and 170 Hz for the β-conformation) is neglected.

The signals of all five monosaccharides can be identified and integrated. Table 1 shows the relative intensities of these 10 signals, the ratio between α and β conformation for each monomer, and the abundance of each monomer in EMn. The ratios of the two conformations correspond well to the table values within the range of uncertainty: as example, according to the ^13^C DEPT intensities, galactose is found to be to 31% in α- and 69% in β-conformations of the pyranose form, in good agreement with the known percentages of 32% and 64%, respectively, in aqueous solution (the about 4% furanose form is ignored here). Overall, galactose constitutes 36% of the monosaccharides in the studied sample of marennine, followed by xylose and rhamnose with about 20% each and mannose and fucose with about 8% each (Table 1). The complete ^13^C spectrum is shown in Figure S5. The vast majority of higher signals are attributed to these five monosaccharides. Other monosaccharides or other molecules can be present in the hydrolyzed sample in lower concentrations. Furthermore, as there seems to be a certain variation in marennine stemming from different preparations, the numbers in Table 1 might vary.

There is also an N-acetyl signal in the proton spectrum after hydrolysis (1.95 ppm, Figure 2d, × 20.37 ppm ^13^C in HSQC, Figure S1), but no corresponding spin system can be identified. The diffusion coefficient appears to be reduced at that value (Figure S3). In fact, monosaccharides with N-acetyl groups are known to be prone to decomposition during the hydrolysis of polysaccharides [35], and this seems to also be the case here. Independent of that, the 1.95 ppm N-acetyl methyl signal is prominent but overall relatively small, so that the contribution of a corresponding monosaccharide will be rather small.

## 3. Discussion

### 3.1. Extracellular Polysaccharide

The presence of a large amount of this complex polysaccharide inside (apices, IMn) and outside (EMn) the cells brings up the question of its function and its biosynthesis. Chrysolaminarin is present in diatoms in large amounts as energy storage [36], but a relation to marennine, e.g., as carbohydrate source, can be excluded, as it is a glucan. On the other hand, many microorganisms, among them diatoms, are known to produce polysaccharides as major part of their extracellular polymeric substance [37]. The excreted form EMn is part of the extracellular polymeric substance, and from this point of view it is not that surprising that a large part of it is a polysaccharide.

Moreover, exopolysaccharides often have a complex structure, like we find it for marennine, and also the composition of marennine is similar to exopolysaccharides of diatoms, except for the absence (or presence beyond 5%) of glucose. As example, Urbani et al. [38] have found rhamnose, fucose, mannose, and galactose, to a smaller amount xylose and glucose, in the exopolysaccharides of the diatom *Chaetoceros decipiens* by gas chromatography after hydrolysis. Already Haug and Myklestad [37] found a similar composition for extracellular polysaccharides of four *Chaetoceros* species. Additionally, the cell wall and soluble cellular polysaccharides of these and other diatom species consist of these five monosaccharides plus a sometimes significant fraction of ribose and glucose [37,39]. The composition of the glycon part of marennine with these types of monosaccharides is thus consistent with the known monosaccharide production of diatoms.

Underwood and Paterson [26] carried out a meta-analysis of the carbohydrate composition of many extracellular polysaccharide samples in different phases from different diatom species or microphytobenthos assemblages and/or collected on different sites or after cultivation. In that work, the above five monosaccharide are identified as main EPS constituents in most diatom groups, too. An important part of them–but not all–contain also glucose as important component, others uronic acids, and some arabinose in smaller amounts. Interestingly, marennine as galactose-rich and glucose- and arabinose-free polysaccharide would be closest to a sample of colloidal material from mudflats in the bay of Marennes-Oléron (France), the region where oyster greening by marennine has been observed and exploited in aquaculture for a long time [5]. On the other hand, a different study of the composition of EPS produced in the bay of Marennes-Oléron found that colloidal fractions consisted to more than 50% of glucose [40].

The monosaccharide composition reminds qualitatively also to fucoidans, although quantitatively, fucose should be more abundant. Fucoidans would be an interesting candidate, because the negative charges of the sulfates attract metals, which might be responsible for the colour [41]. However, the fucose and the other monosaccharides found after hydrolysis are not sulfated (sulfation would induce a strong ^1^H and ^13^C signal shift on the corresponding site). There is the possibility that sulfates were cut off by the harsh hydrolysis conditions. In the spectra of native marennine, one would expect for α-(1→3) and α-(1→4) linked fucoses [42] sulfated at positions 2, 3 or 4 a series of several relatively high proton chemical shifts (4–5 ppm) [43,44], cross-peaks of which are at least not very abundant. This is in agreement with [29] who can separate a fucoidan fraction from the coloured fraction. Nevertheless, in the view of the complexity of the spectra, a smaller degree of sulfation or sulfation on other sites is not excluded.

### 3.2. Variability

Although the determination of the percentages of the monosaccharides by NMR (Table 1) has a relatively high experimental precision (estimated 3 percentage points), results might vary as a consequence of various factors related to cultivation and extraction/purification (see e.g., for EPS the review of Gügi et al. [39]). In natural environment, polysaccharides also often display a certain variability of their composition and glycosidic linkage. Different preparations of marennine always yield similar, but not exactly the same spectra. In particular, the intensity of the aromatic signals varies a lot (on a low level). However, the key signals of the two-dimensional spectra in this work are very similar to those in [11] (however, in much better spectral quality), although the samples have been prepared in two different laboratories. This shows a certain reproducibility of the main features of the monosaccharide composition and linking as well as of the aliphatic part of extracellular marennine.

### 3.3. Additional Tests

As EMn is a part of the extracellular polymeric substance and full purification seems to have been a critical point in the past [29], care must be taken to verify that the main signals really stem from the molecule that is responsible for the color, and that the EPS are not just a byproduct of imperfect purification. Vandanjon et al. have already shown by ultrafiltration that the colored fraction has a mass in the order of 3–7 kDa [23], while Pouvreau et al. find about 10 kDa by three different techniques [6]–and there is no other contribution of this size in the spectra. We have always found similar spectra containing the key features described above with different preparation techniques, in different laboratories. This counts also for the intracellular form of marennine (IMn). Additional attempts with Solid Phase Extraction (SPE) on a C18 cartridge did not allow for the separation of the polysaccharidic fraction from the color either: while laminarin, taken as a reference, is on the majority eluted with water immediately, the (only) colored fraction is eluted with water/ethanol 50:50. And only this fraction shows the intense NMR signals of carbohydrates.

### 3.4. Chromophore

As polysaccharides are normally not colored, the question of the origin of the blue–green color remains open. In most cases colors are associated with conjugated double bonds or aromatic rings. However, only a very low intensity signal group can be found in the corresponding region of the EMn spectrum (Figure 2b). One reason for this might be extreme line broadening by chemical exchange which has already been observed for aromatics. On the other hand, a high percentage of chromophores per sugar units is not necessary for an intense color. The molar extinction coefficient of marennine is *ε*_672_ = 120,000 cm^−1^ L mol^−1^ [6], thus as high as that of chlorophyll a.

Therefore, the findings in this article are not necessarily in contradiction to the conclusion of Pouvreau et al. [6]; marennine can also be a polyphenol, in its aglycon. Neuville and Daste related–already in 1972–marennine to anthocyanins, however, these are usually small. Apart from anthocyanidins, many other classes of molecules consisting of aromatic rings are known to form glycosides. Other, non-covalent bonding of aromatic dyes to certain nonionic polysaccharides has been investigated by Blackburn [45]. Under certain circumstances hydrogen bonding is possible between the polysaccharide’s hydroxyl groups and either electronegative sites or aromatic rings of the dye [46].

A completely different explanation would be given by metal ions complexed by marennine. If these were paramagnetic, they could even spoil the NMR signals of the complexing environment. It is known that polysaccharides, among them EPS, can attract metal ions [41], and these might be the origin of the color. On the other hand, cyclic voltammetry curves of the redox system identified as being related to a change of the color indicate an exchange of protons and electrons at the same time upon redox reactions, which is rather compatible with a molecule containing quinone groups than with metallic cations [47].

## 4. Materials and Methods

### 4.1. Extraction and Purification of Marennine

The extraction and purification as well as the hydrolysis was repeated many times with varying protocols in order to manifest the correlation between glycosidic NMR signals and the color. Extracellular marennine (EMn) was obtained using two purification procedures associated with two different size cultures of *Haslea ostrearia*: <20 L (small-scale marennine production) as described in Francezon et al. [47], and >100 L (scale-up production) as described by Turcotte et al. [14]. For small-scale production, algae were grown in 40 × 500 mL Erlenmeyer flasks [47], up to the beginning of the plateau phase (2–3 weeks). Then the supernatants containing EMn were pooled and EMn was concentrated by precipitation using chemicals (quick and suitable for lab scale batch, see below). In scale-up production, algae were cultivated in >100 L photobioreactors for about 4 weeks up to the plateau phase [11,14]. For both types of algae production, EMn was purified using filtration and ultrafiltration [47]. Typically, the blue supernatants were filtered a first time using a 12–15 µm porosity, and a second time using 1.2 µm porosity. Regarding intracellular marennine (IMn), it was extracted from algal pellet using mortar and pestle and NH_4_HCO_3_ (250 mM), pH 8, 4 °C in the dark, centrifuged and filtered as described by Pouvreau et al. [48].

The isolation of both EMn and IMn was initially done by ion exchange chromatography as described in [48]. In order to reduce loss and speed up extraction and purification of EM, a different method was introduced: a 1 M NaOH (research grade, Sigma-Aldrich, St. Louis, MO, USA) aqueous solution was added dropwise to the filtrates until complete precipitation of the blue compounds. The precipitate was separated from the colorless solution and dissolved using the minimum amount of a 5% (weight) formic acid solution. This blue acidic solution (Figure 1b) was transferred into a 3.5 kDa MWCO dialysis membrane (Spectra/Por^®^ prewetted tubing, Repligen, Waltham, MA, USA) and dialyzed against distilled water to obtain a concentrated marennine solution.

### 4.2. Hydrolysis

15.7 mg extracellular marennine (EMn) were dissolved in 0.5 M HCl and heated to 95 °C for 2 h. After centrifugation, the supernatant has been lyophilized and resolubilized in D_2_O for NMR studies.

### 4.3. NMR Analysis

Four samples have been studied: (i) a 2 mM solution of EMn in D_2_O, (ii) a 2 mM solution of EMn in H_2_O with 5% D_2_O, (iii) a solution of hydrolyzed EMn in D_2_O, (iv) a 1 mM solution of IMn in D_2_O. NMR spectra of (i) and (ii) have been acquired at 301 K on a Bruker 950 MHz (22.3 T) spectrometer equipped with a 5 mm TCI cryoprobe for ^1^H, ^13^C, ^15^N, and ^2^H (NMR Facility IR RMN THC FR3050, Gif-sur-Yvette, France). The most important experiments for the structural analysis have been: DQF-COSY, TOCSY with 80 ms mixing time, NOESY with 200 ms mixing time, all 2048 increments in the indirect dimension, *t*_1,max_ = 114 ms, 8, 4, and 4 scans, respectively; ^1^H-^13^C HSQC of aliphatic, ring and anomeric region separately for sake of sufficient carbon decoupling, *t*_1max_ between 8 and 17 ms, between 8 and 32 scans. HMBC, HSQC-TOCSY, H2BC with *t*_1max_ between 3.3 and 6.7 ms, between 16 and 64 scans.

A similar set of spectra has been acquired of the hydrolyzed EMn at 301 K on a Bruker 400 MHz (7.0 T) spectrometer equipped with a 5 mm BBFO^+^ probe: DQF-COSY, TOCSY with 70 ms mixing time, both 1500 increments in the indirect dimension, *t*_1max_ = 195 ms, 8 scans. ^1^H-^13^C HSQC, *t*_1max_ = 82.8 ms, 32 scans. DOSY, *δ* = 2 ms, *Δ* = 100 ms, 32 increments, 16 scans. DEPT-135 with 20 K scans.

For signal assignment, the software Sparky [49] and the web tools of CASPER [30] were used.

## 5. Conclusions

Marennine, as analyzed in this work, obtained according to two different purification protocols, is a small heteropolysaccharide of about 10 kDa molecular mass, consisting thus of the order of 50 monosaccharide units. It is polydisperse, and the NMR spectra indicate that its structure is relatively heterogeneous. The most prominent signals stem from β-xylose at the non-reducing end, which indicates a high degree of branching. After hydrolysis, galactose, rhamnose and xylose, and to a less extent mannose and fucose are found. The spectrum of the native marennine shows in addition signals of an N-acetylated amine of unknown type. Aromatic signals are very weak, and there is no other particular indication found to a potential chromophore, the identification of which is subject of an ongoing research project. There are, however, several aglycons consisting of shorter and longer aliphatic groups. It is perhaps this complexity of the structure, a highly branched polysaccharide with aliphatic, probably rather hydrophobic chains, and a–still unknown–chromophore, that has over the years released so many hypotheses on the nature of marennine and covered the access to the polysaccharidic nature.

## Figures and Tables

**Table 1:** Chemical shift, relative intensity of the anomeric (C_1_) ^13^C DEPT signals of hydrolyzed EMn (referred to β-galactose), and fractions of α- and β-conformations according to these intensities compared to their tabular values. Percentages (independent of the conformation) of each of the monosaccharides referred to the total amount of monosaccharides.

Type	^13^C C_1_ (ppm)	Relative Intensity	% Conformation ^1^ (Tabular Value)	Fraction ^2^, α and β
α-Gal*p*	92.22	0.44	31 (32) %	36%
β-Gal*p*	96.39	1.00	69 (64) %	
α-Xyl*p*	92.17	0.27	34 (35) %	19%
β-Xyl*p*	96.56	0.51	66 (65) %	
α-Man*p*	93.97	0.20	62 (67) %	8%
β-Man*p*	93.60	0.12	38 (33) %	
α-Rha*p*	93.92	0.45	55 (60) %	20%
β-Rha*p*	93.43	0.36	45 (40) %	
α-Fuc*p*	92.15	0.11	33 (21) %	8%
β-Fuc*p*	96.16	0.23	67 (67) %	

^1^ Difference to 100%: furanoses. ^2^ Difference to 100%: non-assigned signals.

## Data Availability

The data presented in this study are available on request from the corresponding author.

## Supplementary Materials

The following supporting information can be downloaded at: https://www.mdpi.com/article/10.3390/md21010042/s1: Figure S1: Regions of ^1^H-^13^C HSQC of native extracellular marennine (EMn). Figure S2: ^1^H-^13^C HSQC-TOCSY of native EMn. Figure S3: ^1^H DOSY spectra of native and hydrolyzed EMn. Figure S4: ^1^H, ^13^C chemical shifts and *J*-coupling patterns of identified spin systems of hydrolyzed EMn. Figure S5: ^13^C DEPT-135 spectrum of hydrolyzed EMn.
