# Supplementary material for: The Polysaccharidic Nature of the Skeleton of Marennine as Determined by NMR Spectroscopy
Source: Mar Drugs. 2023 Jan 4;21(1):42. doi: 10.3390/md21010042 (PMC9865362; doi:10.3390/md21010042)
Supplement: Supplementary file 1 [file marinedrugs-21-00042-s001.zip › marinedrugs-2091619-supplementary.pdf]

Supplementary material
to

The polysaccharidic nature of the skeleton of marennine as determined by NMR spectroscopy

Ilhem Zebiri, Boris Jaquette, Nellie Francezon, Mickaël Herbaut, Amina Latigui, Sullivan Bricaud, Réjean Tremblay, Pamela Pasetto, Jean-Luc Mouget, and Jens Dittmer

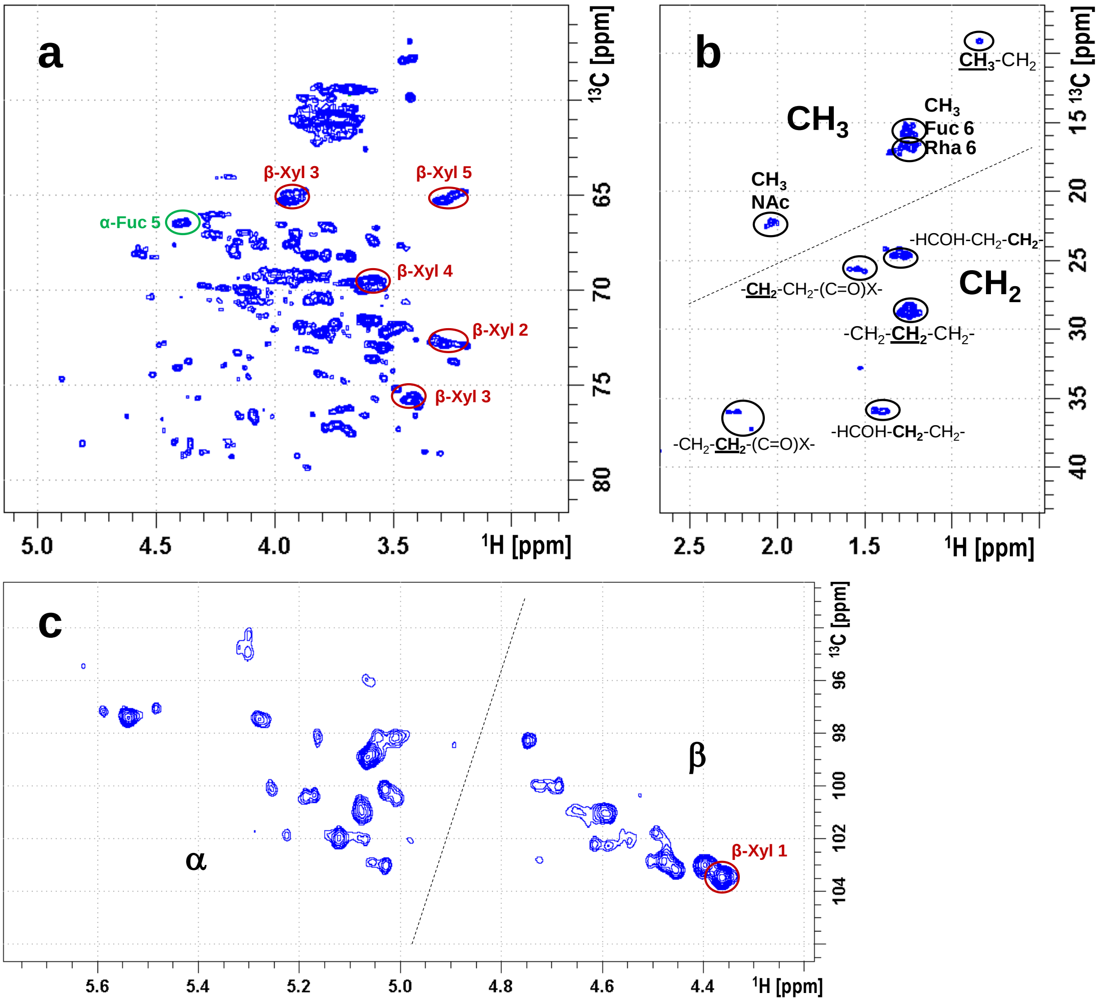

Figure S1. *Regions of ^{1}H-^{13}C HSQC of native extracellular marennine (EMn).* ***(a)*** *ring and CH_2OH,* ***(b)*** *aliphatic,* ***(c)*** *anomeric. Notation: e.g. "5" denotes $C_5 \times H_5$.*

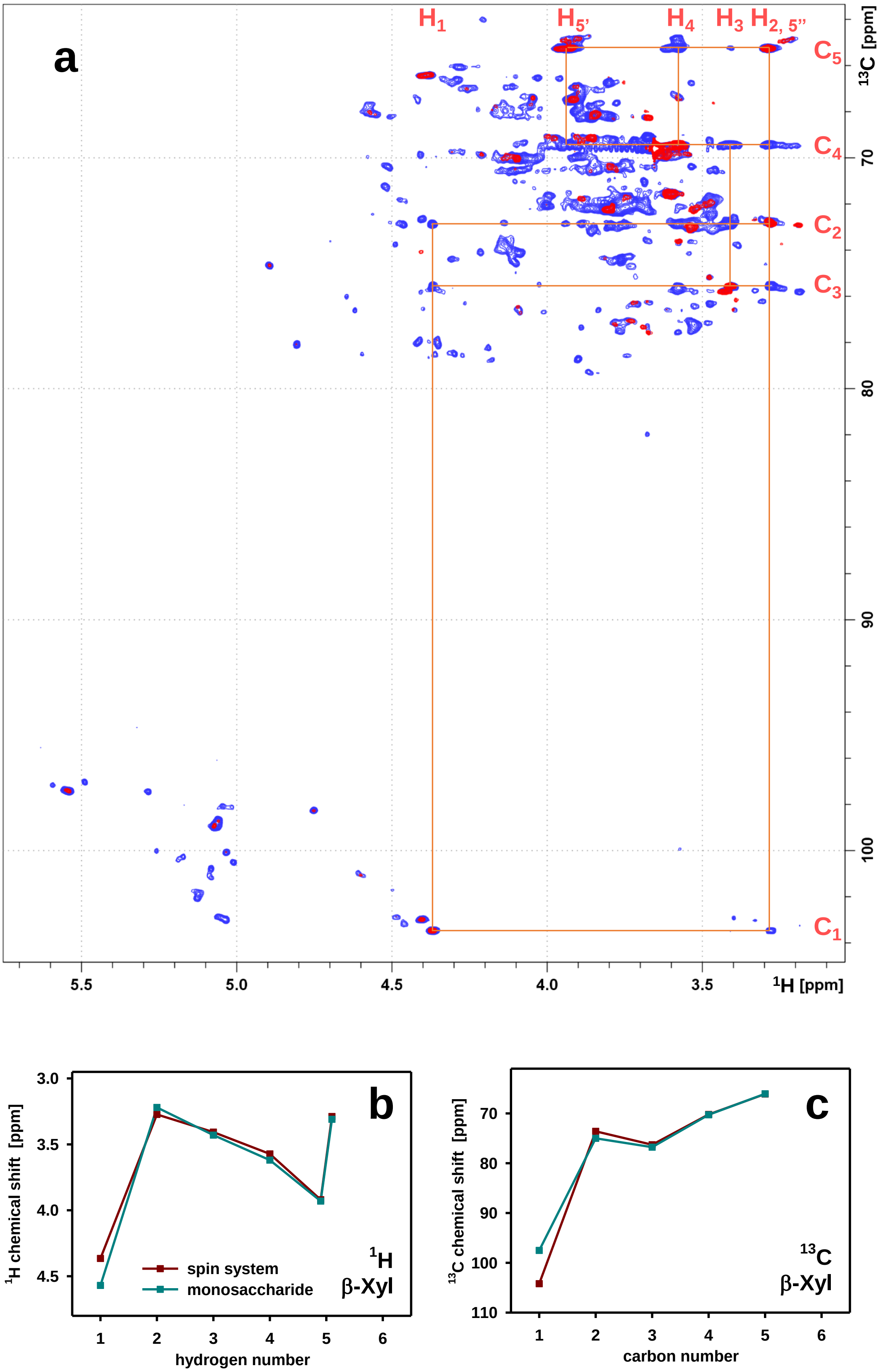

Figure S2. ***(a)*** *Ring and anomeric region of* ^{1}*H-*^{13}*C HSQC-TOCSY of native EMn (blue), superimposed by an HSQC (red). The contour levels are chosen such that only the strongest signals are visible. The lines show the spin system identified as β-xylose*

at the non-reducing end (β-Xyl(1->), as seen by comparison with the ^{1}*H* ***(b)*** *and* ^{13}*C* ***(c)*** *chemical shifts of the monosaccharide (reference values of [30]). In particular the* ^{13}*C shifts show the typical downfield glycosidic bond shift at the anomeric position and upfield at* C_2*, whereas* C_3 *–* C_5 *are unchanged. The experimental shifts have been adjusted (due to different experimental conditions) by +0.8 ppm (see also the monosaccharides part for more details). The lines connect only one central maximum of the partly dispersed signal groups.*

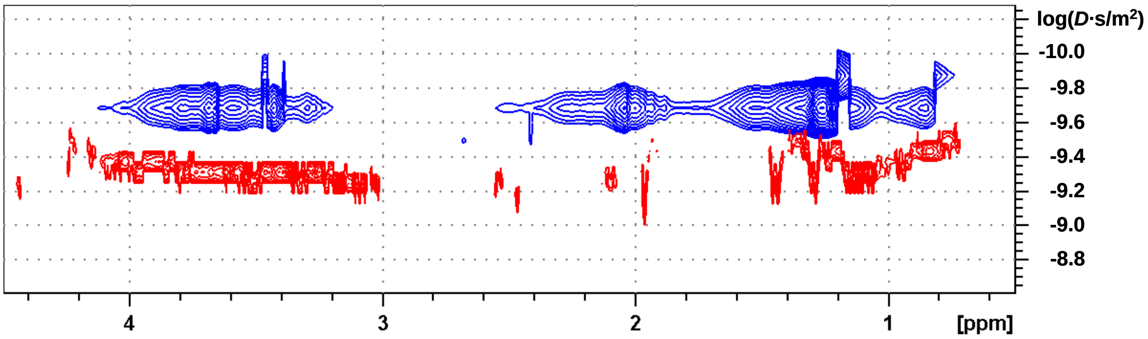

Figure S3. ^{1}*H DOSY spectra of native (blue) and hydrolyzed (red) EMn.*

Xylose

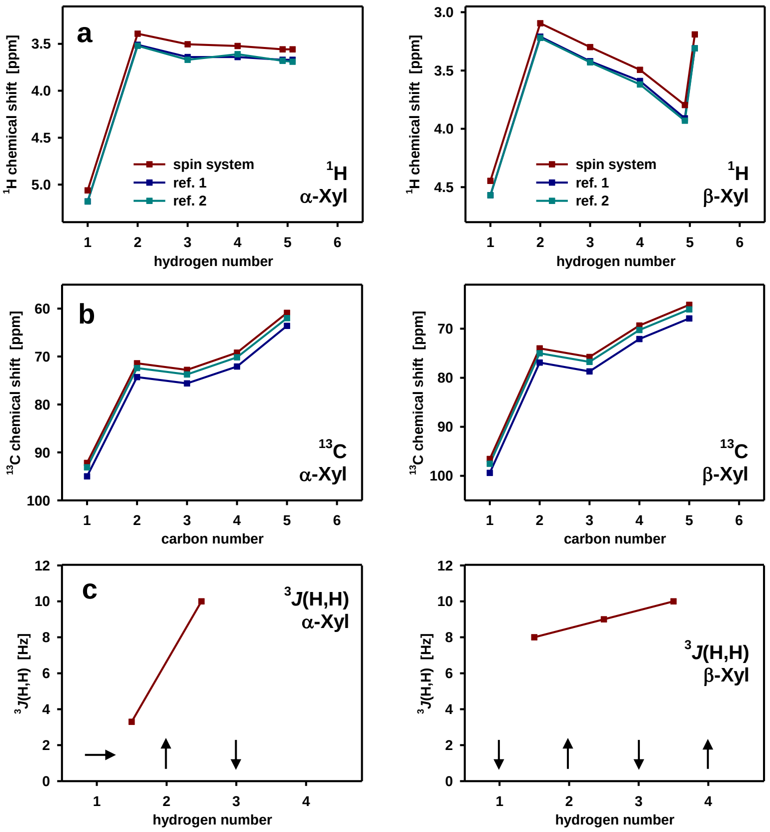

Mannose

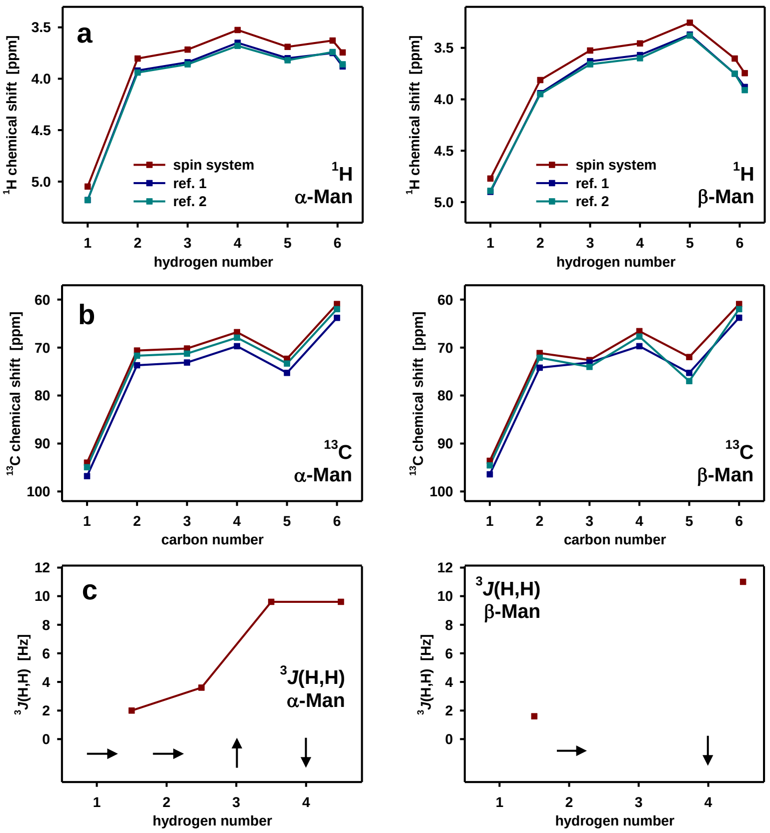

Rhamnose

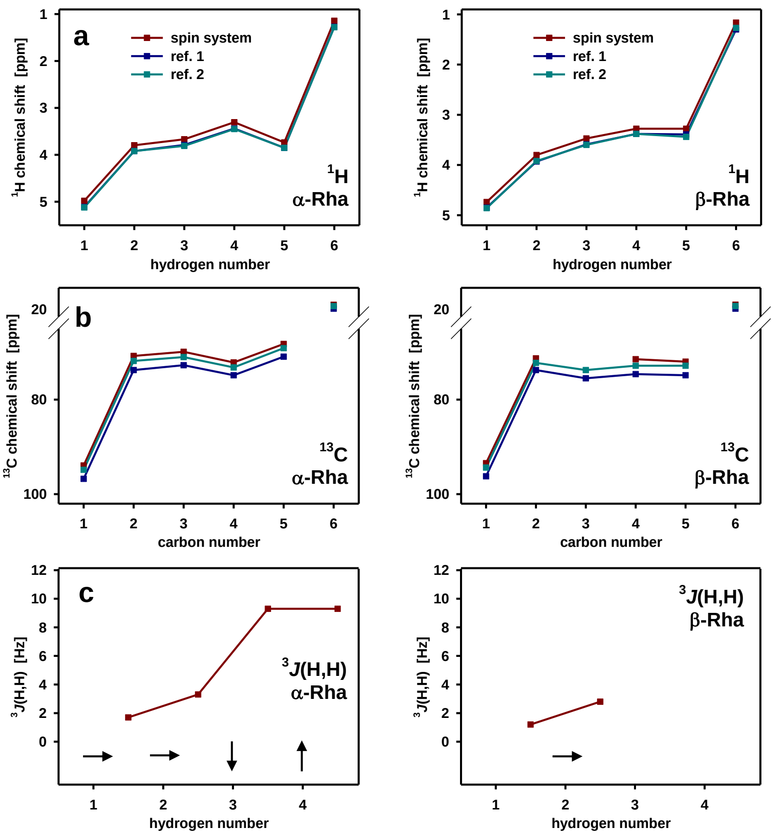

Fucose

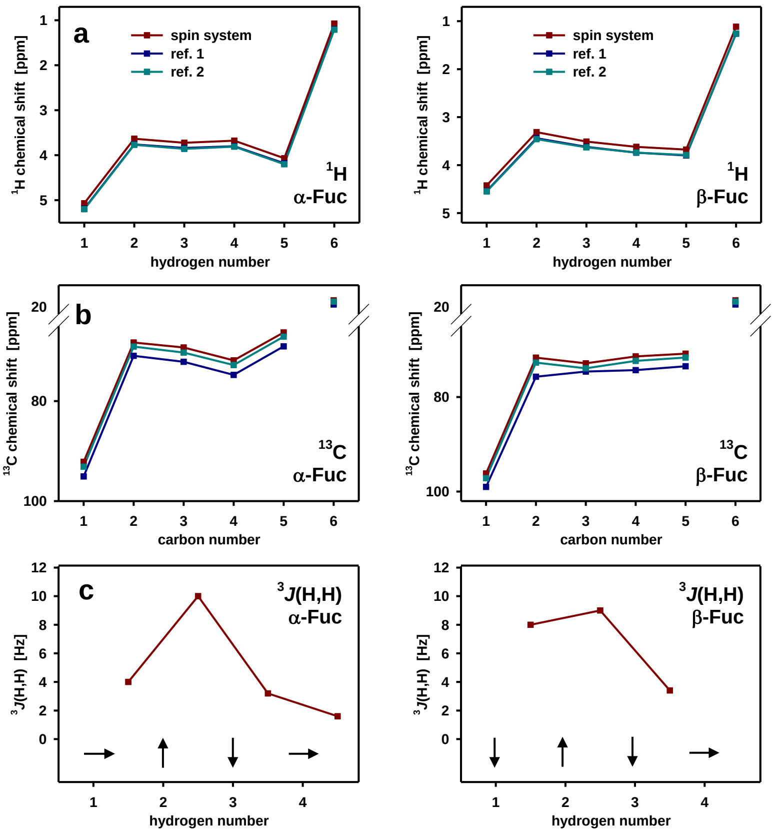

Figure S4. ***(a)*** *^{1}H and* ***(b)*** *^{13}C chemical shifts of identified spin systems of hydrolyzed EMn (red) compared with literature vales of xylose, mannose, rhamnose, and fucose taken from [34] (ref. 1, blue) and [30] (ref. 2, green).* ***(c)*** *J-couplings between neighboured hydrogens as estimated from COSY peak patterns and hydrogen orientation derived herefrom. For galactose, see Figure 4 in the main text.*

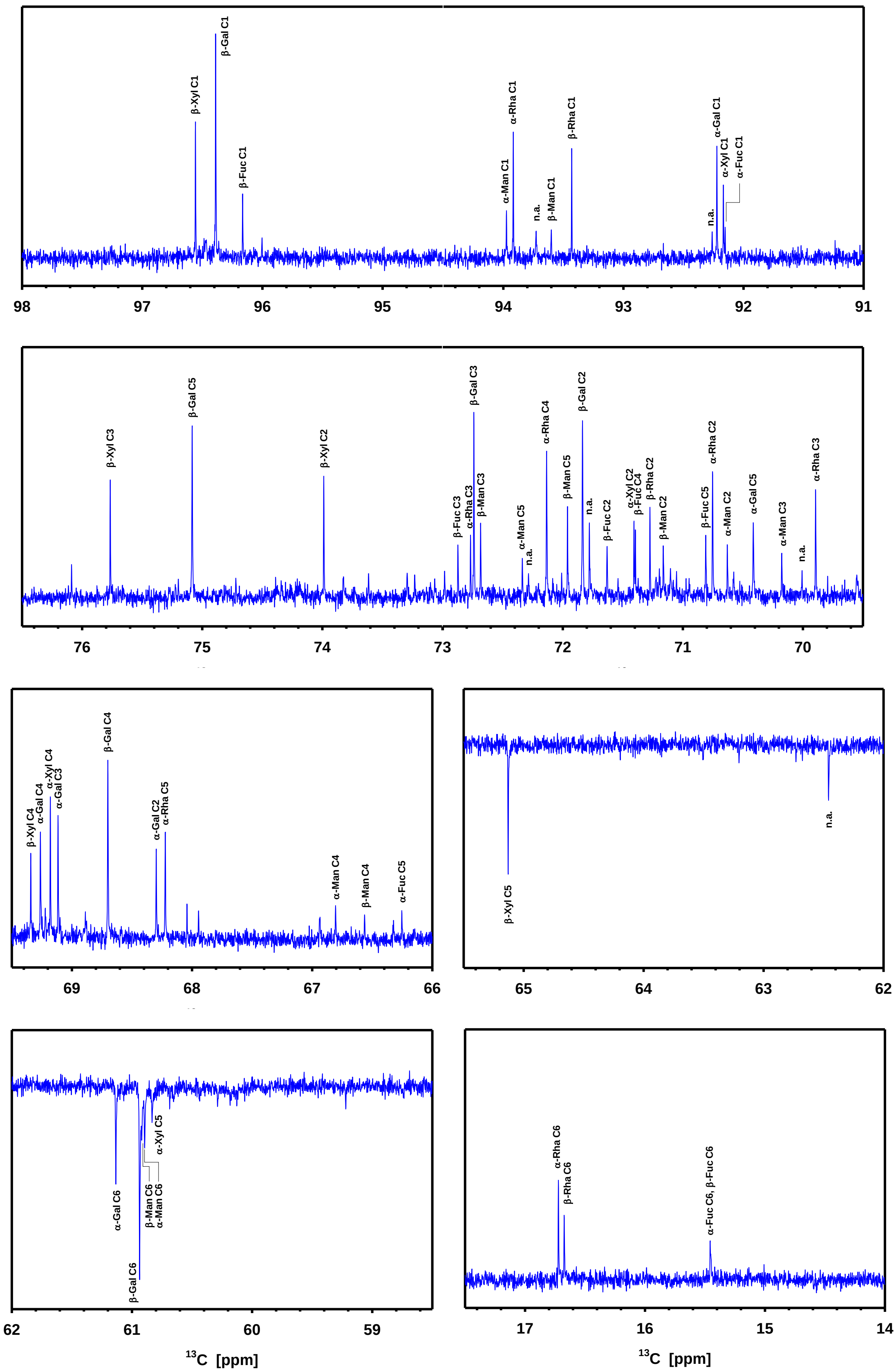

Figure S5. *^{13}C DEPT-135 spectrum of hydrolyzed EMn (n.a.: not assigned).*
